# Automating crystallographic structure solution and refinement of protein–ligand complexes

**DOI:** 10.1107/S139900471302748X

**Published:** 2013-12-25

**Authors:** Nathaniel Echols, Nigel W. Moriarty, Herbert E. Klei, Pavel V. Afonine, Gábor Bunkóczi, Jeffrey J. Headd, Airlie J. McCoy, Robert D. Oeffner, Randy J. Read, Thomas C. Terwilliger, Paul D. Adams

**Affiliations:** a Physical Biosciences Division, Lawrence Berkeley National Laboratory, Berkeley, CA 94720-8235, USA; b Department of Haematology, University of Cambridge, Cambridge Institute for Medical Research, Wellcome Trust/MRC Building, Cambridge CB2 0XY, England; c Los Alamos National Laboratory, Los Alamos, NM 87545-0001, USA; d Department of Bioengineering, University of California at Berkeley, Berkeley, CA 94720-1762, USA

**Keywords:** protein–ligand complexes, automation, crystallographic structure solution and refinement

## Abstract

High-throughput drug-discovery and mechanistic studies often require the determination of multiple related crystal structures that only differ in the bound ligands, point mutations in the protein sequence and minor conformational changes. If performed manually, solution and refinement requires extensive repetition of the same tasks for each structure. To accelerate this process and minimize manual effort, a pipeline encompassing all stages of ligand building and refinement, starting from integrated and scaled diffraction intensities, has been implemented in *Phenix*. The resulting system is able to successfully solve and refine large collections of structures in parallel without extensive user intervention prior to the final stages of model completion and validation.

## Abstract 2

A software system for automated protein–ligand crystallography has been implemented in the *Phenix* suite. This significantly reduces the manual effort required in high-throughput crystallographic studies.

## 1. Introduction   

One of the most important developments in macromolecular crystallography over the past 15 years has been the development of increasingly automated computational tools that are significantly more rigorous and self-diagnostic, thereby decreasing the manual effort involved in structure solution. Besides general improvements in the capabilities of individual components of structure-determination pipelines, especially in the area of automated building (Perrakis *et al.*, 1999 ▶; Cowtan, 2006 ▶; DiMaio *et al.*, 2006 ▶; Terwilliger *et al.*, 2008 ▶), a number of sophisticated pipelines encompassing multiple steps have been described (Bricogne *et al.*, 2003 ▶; Holton & Alber, 2004 ▶; Ness *et al.*, 2004 ▶; Panjikar *et al.*, 2005 ▶; Keegan & Winn, 2007 ▶; Terwilliger *et al.*, 2009 ▶). These projects are especially focused on accelerating the early stages of the process (starting either from raw diffraction images or reduced data), with the goal of obtaining an unambiguous partial model with minimal user intervention. Completing the structure is left to the crystallographer and still remains a largely manual procedure.

In conjunction with these software projects, the productivity of synchrotron beamlines has progressed, driven by a combination of brighter radiation sources (Carwardine *et al.*, 2003 ▶), improved detector hardware (Broennimann *et al.*, 2006 ▶), and automated sample mounting and data collection (Karain *et al.*, 2002 ▶; Cipriani *et al.*, 2006 ▶; Ueno *et al.*, 2006 ▶; Grochulski *et al.*, 2012 ▶). These developments are particularly useful for structure-based drug discovery, which has prompted pharmaceutical companies to build beamlines dedicated to this purpose. Although these structures encompass a relatively small number of target proteins deemed to be of therapeutic utility, the throughput from industrial projects has been estimated at upwards of 10 000 structures per year (Wasserman *et al.*, 2012 ▶). With nearly 600 X-ray crystal structures deposited in the wwPDB (Berman *et al.*, 2003 ▶), the impact of crystallographic studies on the discovery and characterization of the numerous FDA-approved HIV-1 protease inhibitors is undeniable (Wlodawer, 2002 ▶). Similar outcomes with other diseases [*e.g.* hepatitis C virus (Kanda *et al.*, 2013 ▶) and chronic myelogenous leukemia (Milojkovic & Apperley, 2008 ▶)] are on the way to being realised.

An essential task in structure-based drug discovery is the placement of functionally relevant ligands into residual electron density. This has been aided in recent years by a large number of software packages, including *X-LIGAND* (Oldfield, 2001 ▶), *ARP*/*wARP* (Zwart *et al.*, 2004 ▶), *LigandFit* (Terwilliger *et al.*, 2006 ▶), *AFITT* (Wlodek *et al.*, 2006 ▶), *RhoFit* (Global Phasing Ltd) and *PrimeX* (Bell, Cao *et al.*, 2012 ▶). Most ligand-fitting programs incorporate a local real-space refinement step after the initial placement. To varying degrees, most also integrate with ligand-parameterization, model-building and refinement software. However, the very repetitive workflow of high-throughput co-crystallography generally remains a series of discrete steps. Although pharmaceutical companies often develop proprietary internal pipelines (Kroemer *et al.*, 2004 ▶; Mooij *et al.*, 2006 ▶; Davies & Tickle, 2012 ▶; Wasserman *et al.*, 2012 ▶), and several independent groups have automated parts of the process (*e.g.* Tsai *et al.*, 2013 ▶; Sharff *et al.*, 2012 ▶), most of these systems are either integrated with beamline automation or are not readily available to the broad crystallographic community.

While many who have been tasked with solving a large number of crystal structures have developed some means to solve the *n*th structure faster and more easily than the previous *n* − 1 structures out of sheer necessity, the task of developing a truly generic and robust pipeline is more difficult than might at first be imagined. It can be relatively straightforward to optimize a pipeline for one class of structures; however, to make it sufficiently robust to handle very different classes of structures and different qualities of crystallographic data is non-trivial. Consistent with the Pareto principle (Juran & Gryna, 1988 ▶), or the 80–20 rule, much of the development effort remains dedicated to making a small number of cases work. This disparity between effort and percentage success can be explained by the observation that in the course of a structure determination, the crystallographer must make numerous decisions. Many of these decisions rely on his or her experience and are difficult to codify, especially when a program is restricted to only the current coordinate and diffraction data. Even crystallographic steps that are often taken for granted (*e.g.* space-group determination and molecular replacement) are difficult to automate universally because many parameters (*e.g.* solvent content) are only guidelines and because of the pervasive extent to which prior knowledge is naturally and unconsciously utilized.

Here, we describe an integrated pipeline for protein–ligand structure determination as part of the *Phenix* suite (Adams *et al.*, 2010 ▶) that was written to specifically target these historically difficult steps. This pipeline was constructed based on several previously described programs that were intentionally designed around a common framework with future automation in mind (Terwilliger *et al.*, 2006 ▶; McCoy *et al.*, 2007 ▶; Moriarty *et al.*, 2009 ▶; Afonine *et al.*, 2012 ▶). Considerable effort was made to codify the decision-making steps used by experienced crystallographers as they weighed intermediate results against the relevant guideline(s). Starting from processed data, a closely related molecular-replacement search model and basic chemical information about the target ligand, the program is capable of producing high-quality and nearly complete structures with minimal user intervention in many cases. Integrated validation tools (Chen *et al.*, 2010 ▶) assist the user with quality control and completion of the resulting structures. The pipeline was benchmarked against several collections of structure-based drug-discovery protein–ligand complexes and a representative sample of the Iridium database of curated protein–ligand structures (Warren *et al.*, 2012 ▶). In order to ensure no advantage from hindsight, the pipeline was given the same search model and structure factors as used for the published structure determination. The pipeline is able to solve many ligand-bound structures and in some cases can produce results that rival, if not exceed, those of the original deposition.

## 2. Methods   

### 2.1. Basic design   

**Figure 1:**
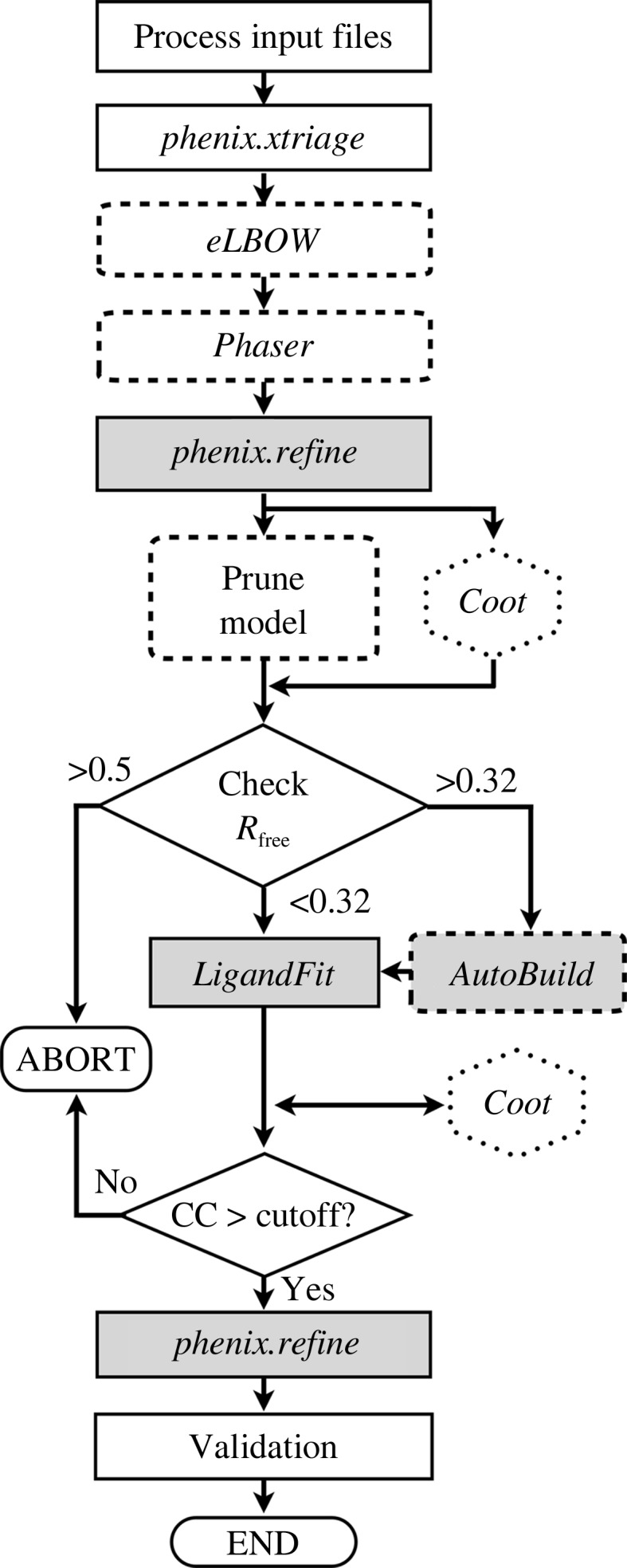
A schematic of the pipeline workflow. Optional modules are highlighted with dashed borders and multiprocessor-aware modules are designated by gray shading. The *Coot* steps are only invoked in interactive mode.

The software (*phenix.ligand_pipeline*) is implemented in Python and is designed to be entirely self-contained within the *Phenix* suite with no external dependencies. An overview of the steps taken is shown in Fig. 1 ▶. The individual steps were encapsulated in modular code so they could be used iteratively and in different workflows. The approach could be extended in the future to adopt a more general-purpose automation framework where components could be removed or added (Tsai *et al.*, 2013 ▶).

In most cases, the program runs with minimal configuration. The only mandatory inputs are processed data (scaled intensities or amplitudes, in any commonly used format), a starting model for molecular replacement (or, if isomorphous, molecular substitution) and a source of ligand geometry information such as a SMILES string or file (Weininger, 1988 ▶), a MOL2 or restraints CIF file, or a PDB residue code that directs *eLBOW* (Moriarty *et al.*, 2009 ▶) to use the entry in the Chemical Components Dictionary (Henrick *et al.*, 2008 ▶). If a directory path is given as input, the program attempts to automatically determine the appropriate input files by scanning the directory contents. Although the processing of raw diffraction images is currently outside the scope of *Phenix*, the program could be extended to run in conjunction with existing automated data-processing pipelines (Winter, 2010 ▶; Vonrhein *et al.*, 2011 ▶).

#### 2.1.1. Data setup and analysis   

An initial step converts the diffraction data to amplitudes in MTZ format as necessary. *R*_free_ flags are either imported or generated if absent. Following this conversion, the data quality is assessed using *phenix.xtriage* (Zwart *et al.*, 2005 ▶) to identify possible twinning and to determine a suitable resolution cutoff if desired. If the number of MR search copies is not defined, it is estimated based on the Matthews coefficient.

#### 2.1.2. Molecular replacement   

Although many ligand co-crystal structures are effectively isomorphous with the native structure and/or each other, the procedure runs *Phaser* (McCoy *et al.*, 2007 ▶) by default to ensure correct placement of the protein. The search model is modified by *Sculptor* (Bunkóczi & Read, 2011 ▶) to match the input sequence as closely as possible, without completing missing side chains; common modified amino acids such as phosphotyrosine are left in place if in agreement with the sequence. The default settings for the MR_AUTO mode are used, except that non-water heteroatoms present in the search model are retained at full occupancy. If desired, the MR solution can be mapped to the same frame of reference as an isomorphous structure using *phenix.find_alt_orig_sym_mate* (Oeffner *et al.*, 2012 ▶).

#### 2.1.3. Ligand generation   

If the input ligand information does not contain full geometry restraints, the molecular geometry is calculated by *eLBOW* and output as restraints in CIF format, coordinates in PDB format and Python pickle files. Currently, the desired stereoisomer must be explicitly requested in the case of chiral ligands; although *eLBOW* is capable of enumerating chiral centers, discrimination between enantiomers will require additional computational decision-making as part of the fitting procedure. Although the default optimization is usually sufficient for ligand placement, the semi-empirical AM1 quantum-mechanical method is also available and may yield improved geometries and parameters.

#### 2.1.4. Initial refinement and rebuilding   

Once the model is correctly placed, *phenix.refine* (Afonine *et al.*, 2012 ▶) is run using the individual coordinate (in both real and reciprocal space) and atomic displacement parameter (ADP) refinement strategies. If *Phaser* was not run previously, rigid-body refinement will be performed with each protein chain as a separate group. A resolution-dependent parameterization is used for determining the ADP type and several other options, including automated rotamer fitting and solvent updating. Simulated annealing is available as an option. The user may also specify custom settings in a parameter file to be passed to *phenix.refine*. Weight optimization is not normally used at this stage, as rapid convergence is considered more important than obtaining an ideal geometry and minimizing overfitting.

Following the initial refinement the model is further processed to remove atoms that may interfere with ligand binding, including waters and side chains with poor fit to density. If the *R*_free_ is greater than a specified cutoff after refinement, indicating severely misfitted regions beyond the radius of convergence of refinement, the *AutoBuild* wizard (Terwilliger *et al.*, 2008 ▶) is used to apply a more aggressive strategy for improving the model (with the default rebuild-in-place mode, which will preserve the input atoms). We have found empirically that an *R*_free_ cutoff of 0.32 is appropriate in most cases, but this can be adjusted by the user.

#### 2.1.5. Ligand fitting   

The *LigandFit* wizard (Terwilliger *et al.*, 2006 ▶) is currently used for placing the target ligand (without H atoms) in the *mF*_o_ − *DF*_c_ map calculated with waters removed, using the geometry specified by *eLBOW*, which produces both the restraints and coordinates in an efficient manner. The difference map may optionally be improved using an automated maximum-entropy procedure (Gull & Daniel, 1978 ▶), which has the effect of extending it to higher resolution; however, by default maps are truncated at 1.5 Å, since the additional detail beyond this was found to not be beneficial (and occasionally to be detrimental) for ligand fitting owing to lower correlation coefficients even when the placement was correct. The number of ligand copies to search for is assumed to be the same as the number of copies of the search model, although this also may be specified by the user. The pipeline uses a slightly more rigorous, but slower, set of options than the settings for the default *LigandFit* procedure to ensure comprehensive sampling of conformations. A cutoff of 0.7 for the correlation coefficient of the ligand to the map is required for the placement to be accepted; if the results for multiple copies are inconsistent, only the highest-scoring ligands are kept. *LigandFit* will use NCS relationships to place ligands if possible, but still filtered by the correlation coefficient of the density fit. A post-processing step follows this with more aggressive treatment of the model, removing clashing protein atoms if a ligand copy generated from NCS operators agrees with the 2*mF*_o_ − *DF*_c_ map. The pipeline is designed to easily accommodate alternative methods of ligand placement (*e.g.* guided ligand replacement; Klei *et al.*, 2014 ▶).

#### 2.1.6. Final refinement   

If at least one copy of the ligand can be placed successfully, a second round of refinement is run with the following more conservative optimization strategy. If the resolution is worse than 1.75 Å, a grid search is used to determine the optimal weight for the X-ray and stereochemistry/*B*-factor terms (Afonine *et al.*, 2011 ▶). Prior to this, any amino acids with missing side-chain atoms owing to pruning or mutations made by *Sculptor* can optionally be automatically rebuilt and refined as an additional step. The placement of elemental ions (Echols *et al.*, in preparation) is also offered as an option for the refinement step. Heteroatoms are sorted and grouped with the nearest chain (similar to structures deposited in the PDB).

Although the models that reach this stage are typically of high quality and near convergence, user intervention becomes unavoidable. Poorly fitted regions of the structure are usually beyond the radius of convergence of simple minimization and require manual rebuilding, and in many cases additional ligands from the buffer or crystallization conditions or missing protein residues may need to be added. As elsewhere in *Phenix* (Echols *et al.*, 2012 ▶), the entire process is integrated with validation and visualization tools to streamline and encourage careful inspection of the structure. In particular, although the overall correlation coefficient is generally a reliable indicator of whether the ligand is in the correct position, the individual molecules still need to be inspected and, if necessary, corrected to verify good agreement with the electron density and prior chemical knowledge, as small local errors may be present. Following refinement, the final model is validated using the *MolProbity* suite (Chen *et al.*, 2010 ▶) as implemented in *Phenix* and a script to view the results in *Coot* (Emsley *et al.*, 2010 ▶) is generated. A simple summary file in the output directory lists each placed ligand and its fit to the electron density as judged by several metrics including overall CC and difference map peaks, with a warning if ligand placement was not entirely successful or if the density metrics suggest (partial) misfitting.

### 2.2. Error handling   

The pipeline terminates at several logical points if relevant quality thresholds are not satisfied. If the *R*_free_ after the initial refinement is above 0.5, indicating an incorrect or incomplete MR solution or a model outside the normal range of convergence, no further building or ligand placement is performed. The cutoff of 0.7 for the ligand–map CC minimizes the risk of false positives, which often go undetected even with manual building (Pozharski *et al.*, 2013 ▶). This may exclude some ligands that are in fact largely correct but include disordered fragments or are present at partial occupancy. The individual output files from *LigandFit* are available for manual inspection if desired. The program does not attempt to reinterpret ligand placements that pass the initial cutoff, but a post-refinement validation step calculates statistics *versus* the final maps and alerts the user if any values are suspicious. Our tests (data not shown) indicate that correctly placed ligands usually have a CC with the 2*mF*_o_ − *DF*_c_ map after refinement of at least 0.9; values below this suggest a partial misfit and/or poor density for part of the ligand and values below 0.8 often indicate a false positive.

### 2.3. Interactive mode   

To address the potential limitations of a fully automatic approach, an interactive mode is available which integrates with *Coot* for manual intervention. After the first refinement, *Coot* is opened with the refined model and maps displayed. Additional changes may then be made to the model, after which the user clicks a button to save the new model and continue the pipeline. New difference maps are calculated and passed to *LigandFit*. *Coot* is then opened a second time with a checklist for the individual ligands. Because the associated restraints CIF file is also loaded into *Coot*, errors in the placement can be corrected by torsion-angle rotation and real-­space refinement. Ligands approved by the user are kept regardless of their initial CC from *LigandFit*.

### 2.4. Testing   

Because our goal is to automate existing workflows, we have primarily tested structures from the PDB where the original MR search model is unambiguously annotated (either in the PDB header or in the relevant publication). In the majority of cases we reduced the model to the minimal asymmetric component, using *phenix.xtriage* to automatically estimate the number of copies present in the target structure based on the solvent content resulting from different numbers of copies. For ligand input, we either used the canonical SMILES string specified in the PDB (including exact chirality) or manually generated a restraints CIF file in *eLBOW*. Where necessary, the restraints needed for any additional ligands present in both the search model and published structure were generated using *eLBOW* or *phenix.ready_set*. The deposited structure was used as a reference model for *phenix.find_alt_orig_sym_mate* as described above and as an atom-name template for *eLBOW*, but the model and geometry were not otherwise used at any stage in the pipeline. For comparison, we also re-refined the published structures using the same protocol as the final refinement step of the pipeline. Ligand-atom names were adjusted as necessary to account for differences in the orientation of chemically symmetric rings (such as phenyl groups) when calculating r.m.s.d.s, without altering the chemistry or pose. All structure figures were generated with *PyMOL* v.1.2.

## 3. Results   

### 3.1. Representative cases   

As examples of high-throughput applications of the pipeline, we examined several sets of related structures in detail. Most of these cases have only a single copy of a ligand and a small protein model. Run times for these examples with default settings averaged between 1 and 2 h on a single-processor core on recent AMD or Intel systems.

#### 3.1.1. Factor Xa   

The protease factor Xa has been a popular drug-discovery target, with more than 120 structures in the PDB (usually in complex with anticoagulant drug leads). We selected a set of five related phenyltriazolinone-bound structures (Quan *et al.*, 2010 ▶), all solved at moderate resolution (2.2–2.75 Å). Because one of these, PDB entry 3ffg, does not have a search model defined in the PDB header, we used the search model (PDB entry 1fjs) used for entry 2p16 by the same depositing author (Pinto *et al.*, 2007 ▶). The other four structures were all phased using PDB entry 3ffg as the search model. All of these structures were completed successfully (Table 1 ▶). The only conformational rearrangement required to accommodate the ligands was a rotation of the Asp189 side chain, which is easily accomplished by a combination of gradient minimization and rotamer fitting in *phenix.refine*. The automatically generated structures are very similar to the deposited models, with the exception of a flipped phenyltriazolinone moiety in PDB entry 3kqb.

#### 3.1.2. Thrombin   

A slightly larger set of structures from an academic group is a series of compounds with human thrombin as a model system for studying the role of solvent in ligand binding (Biela *et al.*, 2012 ▶). The structures were completed successfully by the pipeline (Table 1 ▶) using PDB entry 1h8d as the starting model (Skordalakes *et al.*, 2001 ▶). The final models for all but one structure have ligand conformations that are nearly identical to the published models. The exception, PDB entry 3qwc, differs only by a rotation of the terminal moiety, and rerunning the job using the maximum-entropy procedure in map calculations resulted in a correct fit. A number of details are currently left unmodeled in the structures produced by the pipeline: *e.g.* covalently attached *N*-acetylglucosamine (NAG), other small molecules such as phosphate and glycerol, misfitted side chains and alternate conformations. However, a pair of sodium ions present in the published models were automatically built by *phenix.refine* in eight of the structures using a novel identification procedure (Echols *et al.*, in preparation).

#### 3.1.3. HIV-1 protease   

Human immunodeficiency virus (HIV) protease was one of the earliest and most successful targets of structure-based drug discovery (Wlodawer, 2002 ▶). Because of its clinical importance and the rapid mutation of the viral genome, which often leads to drug resistance, more than 500 structures of various forms of the protein have been deposited in the PDB. Inhibitors typically bind in the cleft formed by the dimer, often in two symmetric poses. Because of this behavior and the frequency of mutations that affect binding, it poses a more challenging test case for automation. We tested the pipeline on a pair of mutant forms bound to the inhibitor atazanavir (Klei *et al.*, 2007 ▶). One of these, designated the inhibitor-resistant mutant (PDB entry 2fxe), is similar to the wild-type structure and binds atazanavir symmetrically; this structure was phased using PDB entry 1hvi (Hosur *et al.*, 1994 ▶). The refined structure was subsequently used to phase the cleavage-resistant mutant (PDB entry 2fxd), which binds the inhibitor in a single orientation and exhibits more local conformational differences relative to the wild type.

**Figure 2:**
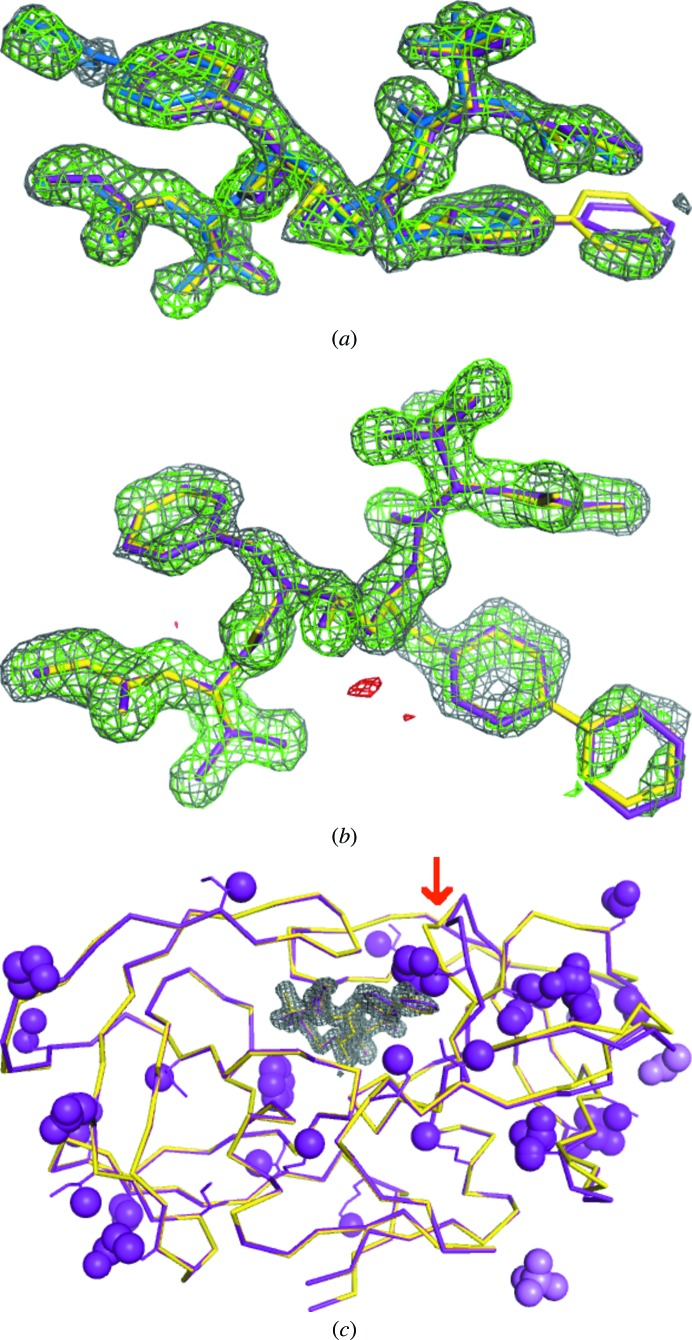
Comparison of refined model and ligand binding between published (purple) and pipeline (yellow) results for atazanavir-bound HIV-1 protease structures (Klei *et al.*, 2007 ▶). The electron density prior to ligand placement is displayed as a gray mesh for the 2*mF*_o_ − *DF*_c_ map (contoured at 1.0σ) and as green and red meshes for the *mF*_o_ − *DF*_c_ map (contoured at ±3.0σ). For clarity, only density within a 1.5 Å radius of the ligand is displayed. (*a*) PDB entry 2fxe; active site of inhibitor-resistant mutant showing published symmetric binding of the inhibitor (with the second conformation colored blue). (*b*) PDB entry 2fxd; active site of the cleavage-resistant mutant. (*c*) Overall structure of the cleavage-resistant mutant at the end of refinement (using chain *A* of 2fxe as the starting model), illustrating the remaining differences in conformation and missing atoms. The red arrow indicates the loop comprising residues 80–83 in chain *B*.

Because the SMILES string for atazanavir in the PDB (residue code DR7) does not specify the chirality of one of the N atoms, we generated the molecular structure manually in *eLBOW*. Although *LigandFit* was able to find both conformations of the ligand in PDB entry 2fxe (*LigandFit* typically finds five candidate placements of a ligand), only the one with the highest CC is selected (Fig. 2 ▶ *a*). Either the protease monomer or the assembled dimer can be used for the input model, with essentially the same outcome. When run with a monomer the pipeline attempts to find two copies of the ligand, but since overlapping placements are not allowed it continues with the single copy and generates a warning at the end of the run. Aside from the lack of a second conformer in PDB entry 2fxe, the automatic ligand placements for both structures are nearly identical to the deposited models (Figs. 2 ▶ ▶ *a* and 2 ▶ *b*). The refined model for PDB entry 2fxe is in very close agreement with the published model and is nearly final aside from some missing side-chain atoms resulting from point mutations. Additional manual work is required to complete the PDB entry 2fxd model (Fig. 2 ▶ *c*); in addition to some incomplete side chains several poorly ordered loops need inspection and possible deletion, in particular residues 80–83 in chain *B*. The optional side-chain completion step is able to restore many of the missing atoms, but the backbone conformation of some residues is sufficiently different to be outside the radius of convergence of the default refinement protocol. Both structures also have several additional unmodeled ligands (acetate, sulfate and glycerol) from the crystallization buffer.

### 3.2. Benchmarking against a diverse test set   

As a more thorough measure of performance, we ran the pipeline on a set of manually curated structures used for testing ligand-docking software (the Iridium-HT test set; http://www.eyesopen.com/iridium), which have been filtered to contain only ligands whose chemical identity is unambiguous with good fit to electron density and no geometrical problems (Warren *et al.*, 2012 ▶). We selected 36 structures representing 31 unique proteins (Supplementary Table S1^1^) for which a single search model can be unambiguously identified from the PDB header. In each case a single ligand species of interest is bound, although some structures also contain additional physiologically relevant ligands that are present in the starting models (such as haem in PDB entry 1g9v or an ATP analog in PDB entry 1hq2). The ligands vary widely in size and structure, from pantoate (C_6_H_11_O_4_) to large drug-like molecules. The pipeline results (Table 2 ▶), when run with default settings, can be summarized as follows.

**Figure 3:**
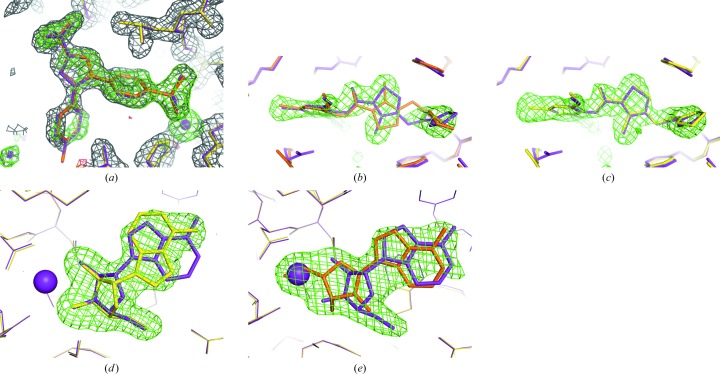
Examples of problematic structures in the Iridium test set. Electron density after the first round of refinement (prior to ligand fitting) is displayed as a gray mesh for the 2*mF*_o_ − *DF*_c_ map (contoured at 1.0σ) and as green and red meshes for the *mF*_o_ − *DF*_c_ map (contoured at ±3.0σ). The published model is shown as purple sticks. Yellow sticks represent the refined model at the end of pipeline execution; orange sticks represent incorrect or rejected ligand placements. (*a*) PDB entry 1oq5. The ligand is correctly placed but is rejected because the CC to the *mF*_o_ − *DF*_c_ map falls below the default cutoff of 0.7. (*b*) PDB entry 1mq6. The ligand is partially misfitted in the run with default settings (orange sticks) owing to ambiguous *mF*_o_ − *DF*_c_ density. Filtering the map with a maximum-entropy procedure results in correct placement (*c*). (*d*) Ligand placement in 1hp0, showing deviation from the published structure. (*e*) Misfitted ligand in 1hp0 with calcium ion (purple sphere) removed from the starting model.

(i) 21 (58%) of the structures (PDB entries 1br6, 1b9v, 1exa, 1fcx, 1fcz, 1hq2, 1hwi, 1k3u, 1ml1, 1n2j, 1of1, 1of6, 1pmn, 1q1g, 1q41, 1r9o, 1tt1, 1w1p, 1w2g, 1yv3 and 2br1) worked unambiguously with default settings and without intervention; manual inspection confirmed that the ligand placement was essentially correct, with only minor disagreements with the published model (if any). For PDB entry 1of6, we used TYR (l-tyrosine) as the target residue based on visual inspection of the deposited model, which is incorrectly labeled as containing DTY (d-tyrosine).
(ii) PDB entry 1g9v ran successfully, but the number of copies of ligands was manually specified because the estimated number of copies of the ligands was incorrect (owing to the use of the complete hemoglobin tetramer as the search model *versus* two ligands bound).
(iii) PDB entry 1l2s also ran successfully, but a third copy of the ligand bound between the two monomers was not built. Both active-site ligands were placed identically to the published structure, but the search for the third failed owing to the interference of a reoriented Gln side chain in the search model.
(iv) The pipeline initially failed to solve PDB entry 2ack owing to the number of copies of the protein being estimated incorrectly, resulting in an *R*_free_ above the cutoff for contin­uing; re-running with this explicitly specified was successful without further intervention.
(v) The pipeline also failed on PDB entry 1oq5 owing to a poor CC for the ligand density, despite nearly perfect placement (Fig. 3 ▶ *a*). Re-running with a more permissive CC cutoff of 0.6 was successful.
(vi) PDB entry 1mq6 runs to completion, but one section of the ligand was misfitted owing to ambiguous difference density (Fig. 3 ▶ *b*). A second run with maximum-entropy map treatment improved the density enough to result in successful placement (Fig. 3 ▶ *c*).
(vii) Two structures, PDB entries 1fjs and 1unl, were both unsuccessful in the initial run of the pipeline but could be recovered using the maximum-entropy map calculation (with the exception of an omitted phenyl ring with poor density in 1unl).
(viii) Two structures, PDB entries 1mzc and 1u4d, finished without error with one or more ligands placed at the expected site(s) but either failed to place all copies requested or had significant errors in the ligand conformation, geometry or orientation upon visual inspection. Some of these were easily remedied with minor adjustments in *Coot*.
(ix) PDB entry 1hww failed because the ligand (swainsonine, residue code SWA) consists of a flexible double-ring system that needs to be nonplanar to correctly fit the density, a degree of freedom not currently explored by *LigandFit*.
(x) Four structures (PDB entries 1cx2, 1qhi, 1yqy and 4cox) required more extensive rebuilding of the placed search model before ligand placement can be successful and were aborted early.

In summary, these tests indicate that use of the pipeline with default parameters is likely to be successful in a high percentage of cases (more than 50%), while the adjustment of one or more parameters may be required for optimal success in another 30% of cases. The failure rate owing to pathological problems with ligand structure or large structural differences between the search and final model is surprisingly small at 15%.

As the intention of the pipeline is to solve, fit and refine protein–ligand complexes, the output models are not publication-ready and require varying degrees of intervention to replace missing or mutated side chains, rebuild loops or place additional ligands. In PDB entry 1r9o, for instance, the search model (PDB entry 1n6b) has only 76% sequence identity and although ligand placement was successful, there are large regions on the surface of the protein that have undergone significant conformational changes and require rebuilding or deletion. However, in ten cases the final *R*_free_ for a successful run was within 1% of the re-refined deposited structure and the geometry quality was consistently high, with a *MolProbity* clashscore (Chen *et al.*, 2010 ▶) in the single digits for all successfully completed runs. For many of the examples, it is likely that an alternative search model is now available that more closely resembles the crystallized conformation and would significantly improve convergence; however, we restricted our tests to using the original search models specified by the authors.

In most structures in which ligand placement is successful, the majority of the runtime is accounted for by refinement, particularly when running weight optimization (Supplementary Table S2). Running *Phaser* has a relatively small impact on the overall runtime, since most MR solutions are unambiguous (and in most of the test cases there was only a single component to place). *LigandFit* is typically the next most time-consuming step and this time scales with the number of copies of the ligand. Because both *phenix.refine* and *LigandFit* can use multiple processor cores on Linux and Macintosh systems, the elapsed runtime can be significantly shortened on multi-core systems. However, for large sets of similar structures such as those discussed in the previous section, processing multiple data sets in parallel with a single processor per job may be a significantly more efficient use of computing resources. If desired, the execution time may be reduced by disabling weight optimization or by running *LigandFit* in ‘quick’ mode, at the expense of potentially poorer output model quality and possible failure of ligand placement.

## 4. Discussion   

The procedure described here has been exercised on hundreds of structures in the PDB (data not shown) with the goal of ensuring robust behavior regardless of the ultimate outcome. Owing to the conservative criteria for evaluating the *LigandFit* results, the number of false positives (where a ligand is placed in the wrong site) has proven to be very low when used with default parameters. In favorable cases, where the crystallized protein has minimal changes relative to the starting model, the final structure is very nearly complete and can easily be finalized by a single round of manual inspection/correction and refinement. In several tests, the *R*_free_ was lower than the published structures. Although this reduction is likely to be partially owing to improvements in refinement protocols and/or under-refinement of the original models (Joosten *et al.*, 2009 ▶; Afonine *et al.*, 2012 ▶), it does demonstrate the ability of an automated pipeline to produce relatively high quality structures. However, we also encountered situations that are challenging for automation and potentially also for manual analysis.

Even if the model is extremely accurate and complete, limitations in map quality can hamper automatic identification of the correct binding site. In some examples (such as PDB entry 1oq5 in the Iridium test set), *LigandFit* places the ligand(s) correctly but the pipeline rejects these models owing to a poor CC to the difference map. Alternatively, the presence of additional unmodeled blobs of difference density may be fitted preferentially, although such false positives are usually also rejected based on the CC. These limitations on sensitivity may make the pipeline less optimal for fragment-based drug discovery, where the ligands are typically smaller and bind with lower affinity (and partial occupancy). More flexibility may be required in the ligand-fitting step for these structures, such as fitting to the 2*mF*_o_ − *DF*_c_ map and using a more sensitive metric than the CC. However, we found the use of maximum-entropy maps to be very helpful for several of the test cases, as it effectively increases the resolution of the Fourier map and eliminates the bulk of noise (Collins, 1982 ▶). Although the pipeline can be run in a more permissive mode by decreasing the CC cutoff and/or searching for more copies of the target ligand, this is not guaranteed to place weakly defined ligands correctly, as the presence of additional unmodeled density (for protein or other buffer components) may frustrate the fitting procedure.

More generally, the use of relatively simplistic geometry restraints instead of a physically realistic force field may limit the accuracy of ligand placement in ways that are not easily detectable by automated procedures. In particular, although the refinement is performed with explicit H atoms, the lack of attractive forces or solvation effects may miss fine chemical detail such as hydrogen bonding and hydrophobic inter­actions. The use of molecular-mechanics force fields for crystallographic refinement has been shown to yield improved protein geometry in some cases (Koparde *et al.*, 2011 ▶; Schnieders *et al.*, 2011 ▶; Bell, Ho *et al.*, 2012 ▶) and it may help to overcome limitations inherent to low-resolution data sets. Refinement against a quantum-mechanical potential may also produce more accurate geometry (Li *et al.*, 2009 ▶).

In our tests, the most common reason for failure of ligand placement was the presence of large conformational differences from the true structure, even after the first cycle of refinement. In many structures, such as protein kinases, significant conformational changes on the scale of small loops (*e.g.* P-loop, DFG loop, activation loop) to entire domains (*e.g.* the N-terminal lobe) accompany ligand binding. These movements are usually outside the range of advanced refinement protocols, such as simulated annealing and the deformable elastic network method (Schröder *et al.*, 2007 ▶; Brunger *et al.*, 2012 ▶), and instead require extensive rebuilding. Although misfitted residues can be removed from the model, aggressive pruning often results in ligand placement attempting to utilize the difference density for the removed protein atoms rather than only focusing on the true binding site. Because the current approaches to automated model building in *Phenix* (Terwilliger *et al.*, 2008 ▶) are aimed at either *de novo* building into an experimental map or minor changes to an existing model, we have not made extensive use of them in the context of the pipeline. However, targeted application of loop-fitting methods and inference from related structures may overcome the rebuilding problem without greatly increasing the runtime. It is also likely that many structures can be solved more effectively by automatically testing multiple search models in molecular replacement (Keegan & Winn, 2007 ▶; Long *et al.*, 2008 ▶; Bunkóczi *et al.*, 2013 ▶). The large numbers of PDB entries closely related to most popular drug targets offer an additional source of structural diversity that could be utilized in rebuilding.

In challenging cases, use of the interactive mode can efficiently help address problems. Such cases are readily identified after a first round of automated use of the pipeline. Additionally, for a series of related compounds, once the structure of the first protein–ligand complex has been solved, it can be used as the starting model for the remainder in an automated manner. Such was the case with the factor Xa structures (Quan *et al.*, 2010 ▶) presented earlier. Irrespective, manual inspection of the pipeline results (a step that is streamlined by the generation of a *Coot* script after refinement) is required to determine the next steps for structure completion. A final round of careful validation, remediation of outstanding model deficiencies and refinement is essential before publication or deposition. Generally, further improvements in structure completion (*e.g.* local model rebuilding, modeling of alternate conformations, placement of small ions and additional ligands) are needed to enable researchers to generate deposition-ready structures in a fully automated manner. The adoption and diligent use of robust validation tools (Chen *et al.*, 2010 ▶; Read *et al.*, 2011 ▶; Pozharski *et al.*, 2013 ▶) both during and after the structure-determination process will continue to be vital as these approaches become more sophisticated and widespread.

## 5. Availability   

The program *phenix.ligand_pipeline* is distributed with source code in the *Phenix* software suite (http://www.phenix-online.org) version 1.8.3 or later. The complete suite is freely available to academic users.

## Supplementary Material

Supporting Tables S1 and S2.. DOI: 10.1107/S139900471302748X/lv5055sup1.pdf

## Figures and Tables

**Table 1:** Statistics of pipeline runs for selected factor Xa (Quan *et al.*, 2010 ▶), thrombin (Biela *et al.*, 2012 ▶) and HIV-1 protease (Klei *et al.*, 2007 ▶) data sets Unless specified by ‘C’ for custom, default parameters were used. For custom runs, only one attempt was made to adjust the parameters (*e.g.* lowering the CC cutoff for accepting ligand placements) and to get the refinement to complete successfully. A ‘P’ for partial indicates that at least one of the copies of the placed ligand was mostly, but not completely, correct (*e.g.* it was placed at the correct location but one or more torsion angles were not set properly).

Protein	PDB code	*d*_min_ (Å)	Re-refined *R*_work_/*R*_free_	Pipeline *R*_work_/*R*_free_	Ligand r.m.s.d. (Å)
Factor Xa	3ffg	1.54	0.156/0.186	0.159/0.197	0.25
	3kqb	2.25	0.170/0.193	0.175/0.202	1.21
	3kqc	2.20	0.165/0.197	0.172/0.209	0.93
	3kqd	2.75	0.189/0.238	0.202/0.239	0.52
	3kqe	2.35	0.173/0.213	0.182/0.227	0.77
Thrombin	3p17	1.43	0.126/0.157	0.140/0.161	0.93
	3qto	1.52	0.144/0.158	0.156/0.172	0.06
	3qtv	1.63	0.144/0.164	0.153/0.174	0.07
	3qwc	1.74	0.145/0.168	0.152/0.177 P	3.69
				0.151/0.173 C	0.08
	3qx5	1.35	0.123/0.146	0.136/0.153	0.06
	3sha	1.52	0.145/0.169	0.157/0.178	0.08
	3shc	1.90	0.157/0.179	0.162/0.164	0.20
	3si3	1.55	0.142/0.164	0.151/0.175	0.17
	3si4	1.27	0.134/0.155	0.144/0.163	0.07
	3sv2	1.30	0.136/0.163	0.150/0.172	0.09
HIV-1 protease	2fxd	1.60	0.181/0.205	0.216/0.247	0.13
	2fxe	1.80	0.165/0.199	0.179/0.199	0.33†

† Single conformation only.

**Table 2:** Summary of the *phenix.ligand_pipeline* results for the Iridium test set (listed in alphabetical order by PDB code) The re-refined *R*_work_/*R*_free_ values for the deposited models were generated using the same protocol as the final stage of the pipeline. The placed/present column gives the number of copies of the ligand placed out of the number of copies in the asymmetric unit. All copies of the target ligand were successfully placed in the first attempt for 21 of the 36 test cases; another five were successful after minor parameter adjustments. Partial solutions were obtained for six of the problematic cases (designated by italic type).

PDB code	Re-refined *R*_work_/*R*_free_	Pipeline *R*_work_/*R*_free_	Placed/present	Ligand r.m.s.d.(s) (Å)
1b9v	0.175/0.202	0.200/0.224	1/1	0.65
1br6	0.180/0.224	0.192/0.225	1/1	0.20
*1cx2*	*0.245/0.308*	*0.250/0.362*	*0/4*	—
1exa	0.169/0.192	0.182/0.213	1/1	0.06
1fcx	0.135/0.167	0.152/0.181	1/1	0.13
1fcz	0.141/0.175	0.157/0.182	1/1	0.08
*1fjs*	*0.158/0.204*	*0.231/0.255*	*0/1*	—
		*0.178/0.208*	*1/1 C*	*0.59*
1g9v	0.141/0.166	0.135/0.171	1/2	0.17
		0.136/0.171	2/2 C	0.16, 0.17
*1hp0*	*0.163/0.216*	*0.173/0.227*	*2/2 P*	*0.31, 2.04*
1hq2	0.121/0.160	0.134/0.166	1/1	0.10
1hwi	0.165/0.189	0.177/0.198	4/4	0.32–0.44
*1hww*	*0.136/0.167*	*0.222/0.248*	*0/1*	—
1k3u	0.135/0.162	0.137/0.165	1/1	0.05
1l2s	0.147/0.168	0.168/0.195	2/3	0.15, 0.16
1ml1	0.159/0.208	0.173/0.208	6/6	0.25–0.86
*1mq6*	*0.171/0.221*	*0.184/0.227*	*1/1 P*	*1.36*
		*0.179/0.230*	*1/1 C*	*0.68*
*1mzc*	*0.146/0.168*	*0.152/0.168*	*1/1 P*	*2.51*
1n2j	0.168/0.193	0.185/0.210	2/2	0.10, 0.25
1of1	0.156/0.179	0.175/0.197	2/2	0.11, 0.13
1of6	0.173/0.191	0.208/0.230	8/8	0.11–1.82
1oq5	0.120/0.164	0.231/0.264	0/1	—
		0.148/0.188	1/1 C	0.86
1pmn	0.190/0.224	0.218/0.249	1/1	0.53
1q1g	0.156/0.185	0.195/0.214	6/6	0.18–0.63
1q41	0.182/0.195	0.207/0.222	2/2	0.19, 0.30
*1qhi*	*0.214/0.253*	*0.348/0.403*	*0/1*	—
1r9o	0.162/0.193	0.240/0.284	1/1	0.39
1tt1	0.147/0.171	0.158/0.182	2/2	0.17, 0.19
*1u4d*	*0.187/0.206*	*0.200/0.219*	*2/2 P*	*0.64, 0.67*
*1unl*	*0.190/0.214*	*0.264/0.291*	*0/1*	—
		0.246/0.276	1/1 C	0.98
1w1p	0.196/0.230	0.225/0.257	2/2	0.22, 0.30
1w2g	0.177/0.198	0.204/0.221	2/2	0.26, 0.56
*1yqy*	*0.201/0.246*	*0.351/0.411*	*0/1*	—
1yv3	0.151/0.184	0.169/0.193	1/1	0.19
2ack	0.159/0.185	0.446/0.507	—	—
		0.165/0.193	1/1 C	0.42
2br1	0.162/0.195	0.170/0.212	1/1	0.33
*4cox*	*0.205/0.30*	*0.256/0.361*	*0/4*	—

## Footnotes

1 Supporting information has been deposited in the IUCr electronic archive (Reference: LV5055).
