# Supplementary material for: Automating crystallographic structure solution and refinement of protein–ligand complexes
Source: Acta Crystallogr D Biol Crystallogr. 2013 Dec 25;70(Pt 1):144–54. doi: 10.1107/S139900471302748X (PMC3919266; doi:10.1107/S139900471302748X)
Supplement: Supplementary file 1 [file d-70-00144-sup1.pdf]

Supplementary Material

Supplementary Table S1. Selected structures from the Iridium test set arranged by protein. The Ligand column gives the Chemical Component Dictionary (Henrick *et al.,* 2008) code for the ligand to be placed by *phenix.ligand_pipeline*. (* 1OF6 contains DTY in the deposited model, but the chemical entity is clearly TYR.)

Protein	PDB ID	d_min (Å)	Ligand	Reference
Influenza neuraminidase	1B9V	2.35	RA2	Finley *et al.*, 2000
Ricin	1BR6	2.30	PT1	Yan *et al.*, 1997
Cyclooxygenase-2 (COX-2)	1CX2	3.00	S58	Kurumbail *et al.*, 1996
	4COX	2.90	IMN	
	1OQ5	1.50	CEL	Weber *et al.*, 2004
Retinoic acid nuclear receptor gamma-1	1EXA	1.59	394	Klaholz *et al.*, 2000a
Retinoic acid nuclear receptor gamma-2	1FCX	1.47	184	Klaholz *et al.*, 2000b
	1FCZ	1.38	156	
Factor Xa	1FJS	1.92	Z34	Adler *et al.*, 2000
	1MQ6	2.10	XLD	Adler *et al.*, 2002
Hemoglobin	1G9V	1.85	RQ3	Safo *et al.*, 2001
T. vivax nucleoside hydrolase	1HP0	2.10	AD3	Versees *et al.*, 2001
6-hydroxymethyl-7,8-dihydropterin pyrophosphokinase	1HQ2	1.25	PH2	Blaszczyk *et al.*, 2003
HMG-CoA reductase	1HWI	2.30	115	Istvan & Deisenhofer, 2001
alpha-mannosidase II	1HWW	1.87	SWA	van den Elsen *et al.*, 2001
Tryptophan synthase	1K3U	1.70	IAD	Weyand *et al.*, 2002
AmpC beta-lactamase	1L2S	1.94	STC	Powers *et al.*, 2002
Triosephosphate isomerase	1ML1	2.60	PGA	Thanki *et al.*, 1997
Protein farnesyltransferase-geranylgeranyltransferase-I	1MZC	2.00	BNE	deSolms *et al.*, 2003
Pantothenate synthetase	1N2J	1.80	PAF	Wang & Eisenberg, 2003

HSV1 thymidine kinase	1OF1	1.95	SCT	Schelling *et al.*, 2004
	1QHI	1.90	BPG	Bennett *et al.*, 1999
3-deoxy-D-arabino-heptulosonate-7-phosphate synthase	1OF6	2.10	TYR*	König *et al.*, 2004
JNK3	1PMN	2.20	984	Scapin *et al.*, 2003
P. falciparum Purine nucleoside phosphorylase	1Q1G	2.02	MTI	Shi *et al.*, 2004
GSK-3beta	1Q41	2.10	IXM	Bertrand *et al.*, 2003
Cytochrome P450 2C9	1R9O	2.00	FLP	Wester *et al.*, 2004
GluR6 ligand-binding domain	1TT1	1.93	KAI	Mayer, 2005
Cdc42-associated tyrosine kinase ACK1	1U4D	2.10	DBQ	Lougheed *et al.*, 2004
CDK25	1UNL	2.20	RRC	Mapelli *et al.*, 2005
Cyclic dipeptide chitinase	1W1P	2.10	GIO	Houston *et al.*, 2004
M. tuberculosis thymidylate kinase	1W2G	2.10	THM	Fioravanti *et al.*, 2005
Anthrax lethal factor	1YQY	2.30	915	Shoop *et al.*, 2005
Myosin II	1YV3	2.00	BIT	Allingham *et al.*, 2005
Acetylcholinesterase	2ACK	2.40	EDR	Ravelli *et al.*, 1999
Chk1	2BR1	2.00	PFP	Foloppe *et al.*, 2005

Supplementary Table S2. Run times in seconds of individual tasks for three representative structures from the Iridium test set for which *phenix.ligand_pipeline* ran to completion and correctly placed all copies of the ligand in the asymmetric unit. The system used for benchmarking was a 64-core 1.4Ghz AMD Opteron 6376 running Fedora Linux 19. All tasks were run as integrated into *phenix.ligand_pipeline*. The equivalent standalone functionality in *Phenix* is listed below each task. The number of non-hydrogen (*i.e.* heavy) atoms in the structure is given below each PDB ID to provide some sense of relative size. Tasks for which the use of multiple CPUs was beneficial are bolded.

Task	**1BR6**<br>(nproc=1)<br>2,163	**1BR6**<br>(nproc=16)<br>2,163	**1OF1**<br>(nproc=1)<br>5,099	**1TT1**<br>(nproc=1)<br>4,652
Data input	0.1	0.1	0.5	0.6
Data assessment<br>(*phenix.xtriage*)	1.4	1.5	13	7
Molecular replacement<br>(*phenix.phaser*)	47	48	156	78
Ligand parameterization<br>(*phenix.elbow*)	64	68	113	22
Sidechain truncation, water removal, and omit map calculation<br>(*mmtbx.prune_model*)	12	12	78	47
**Ligand fitting**<br>(*phenix.ligandfit*)	270	91	1,599	2,596
**Refinement**[*]<br>(*phenix.refine*)	2,881	1521	12,021	7,197
Validation<br>(Comprehensive validation – *Phenix* GUI)	88	91	431	270
Total	3365	1835	14420	10224

[*]Sum of run times for two separate jobs – one before and one after ligand fitting. As run with default settings, only the second job uses multiple CPU cores (12-16 at a time in the target weight optimization grid search).
